# Therapeutic effects of Bushen Chushi formula on knee osteoarthritis via modulation of MAPK/SLC7A11/GPX4 signaling in rats

**DOI:** 10.1186/s41065-025-00587-1

**Published:** 2025-10-29

**Authors:** Yuanyuan Li, Hai Tang, Mingjiang He, Xinyue Wang, Yanbai Chen, Siye Liu, Hongmei Zhang

**Affiliations:** 1 Arthropathy Dept.1, Wangjing Hospital, China Academy of Chinese Medical Sciences, No. 6 of Wangjing Zhonghuan South Road, Chaoyang District, Beijing, 100102 China; 2 Affiliated Hospital of Shandong University of Traditional Chinese Medicine, Jinan, Shandong Province 250014 China

**Keywords:** Bushen chushi formula, Cartilage, Ferroptosis, Inflammation, Knee osteoarthritis, Subchondral bone

## Abstract

**Objective:**

The aim of this study is to evaluate the therapeutic efficacy of Bushen Chushi Formula (BSCSF) in a rat model of knee osteoarthritis (KOA), with a focus on the modulation of ferroptosis-related signaling pathways and inflammatory responses, and to assess its effects on cartilage integrity and subchondral bone microstructure.

**Methods:**

A total of 66 male Sprague-Dawley rats were randomly assigned to six groups: control, model, diclofenac sodium (DCF), and BSCSF high-dose (BSCSF-H), medium-dose (BSCSF-M), and low-dose (BSCSF-L) groups. KOA was induced via intra-articular injection of monosodium iodoacetate. Following induction, BSCSF or DCF was administered orally for 4 weeks. Therapeutic outcomes were assessed through Lequesne MG scores, body weight measurements, histopathological evaluation (hematoxylin and eosin, Safranin O-Fast Green staining), and micro-computed tomography of the subchondral bone. Expression levels of ferroptosis-associated proteins (SLC7A11, GPX4, p38 MAPK, MMP-13), glutathione (GSH), and iron accumulation (Fe²⁺), as well as inflammatory cytokines (IL-1β, TNF-α), were also measured.

**Results:**

When compared to the model group, rats in the BSCSF-H and BSCSF-M groups exhibited significantly lower Lequesne MG scores, improved cartilage morphology, and enhanced subchondral bone microstructure, as indicated by increased bone volume fraction, trabecular thickness, trabecular number, and connectivity density along with decreased bone surface-to-volume ratio and trabecular separation. These groups also demonstrated increased expression of SLC7A11 and GPX4, elevated GSH levels, and reduced Fe²⁺ accumulation in cartilage, suggesting attenuation of ferroptosis. Inflammatory mediators, including IL-1β, TNF-α, MMP-13, and p38 MAPK, were significantly downregulated, indicating reduced inflammation and extracellular matrix degradation. The BSCSF-L group indicated modest improvements, primarily in inflammatory parameters and joint function.

**Conclusion:**

BSCSF attenuated KOA progression in rats by inhibiting ferroptosis through the MAPK/SLC7A11/GPX4 signaling axis and suppressing inflammatory responses. The observed preservation of cartilage structure and enhancement of subchondral bone quality highlight its therapeutic potential in the management of KOA.

**Supplementary Information:**

The online version contains supplementary material available at 10.1186/s41065-025-00587-1.

## Introduction

Knee osteoarthritis (KOA) is a common chronic degenerative joint disorder characterized by articular cartilage degradation, reactive alterations in subchondral bone, persistent synovial inflammation, and the development of osteophytes along joint margins, collectively leading to a significant reduction in quality of life and, in severe cases, joint dysfunction and immobility [1, 2]. 

Emerging evidence has highlighted the involvement of ferroptosis, a newly recognized form of regulated cell death, in the pathogenesis and progression of KOA. Under mildly acidic conditions, free iron accumulation (Fe²⁺) facilitates the formation of phospholipid hydroperoxides (PLOOH) by donating electrons to intracellular oxygen, which subsequently produces alkoxy radicals via the Fenton reaction [3]. The resulting accumulation of intracellular Fe²⁺ significantly increases lipid peroxidation products, leading to disruption of the cell membrane and eventual cell death.

Glutathione peroxidase 4 (GPX4), the sole enzyme capable of reducing PLOOH to lipid alcohols, plays a key role in preventing their accumulation [4]. As a lipid peroxidase, GPX4 eliminates peroxides embedded in cellular membranes, thereby interrupting the cycle of membrane damage and lipid oxidative stress [5]. The solute carrier family 7 member 11 (SLC7A11), a key subunit of the cystine–glutamate antiporter system, is integral to cellular antioxidant defenses. Upregulation of SLC7A11 promotes the transmembrane transport of cystine, thereby enhancing the synthesis of glutathione (GSH), an essential cofactor for GPX4 enzymatic activity [6–8]. 

Excess intracellular iron also contributes to the generation of reactive oxygen species (ROS) via the Fenton reaction. These ROS further activate proinflammatory signaling pathways, upregulate the expression of inflammatory cytokines and matrix metalloproteinases, inhibit the synthesis of chondrocyte-derived proteoglycans, and induce chondrocyte apoptosis [9, 10]. Thus, ferroptosis not only directly contributes to chondrocyte injury but also amplifies joint degeneration through its regulatory effects on inflammation and MMP expression.

Proinflammatory cytokines are abundantly expressed in the osteoarthritic joint environment and are capable of accelerating cartilage degradation by inducing MMP expression via activation of the mitogen-activated protein kinase (MAPK) pathway. These cytokines also disrupt intracellular iron homeostasis, facilitating the accumulation of free iron and triggering ferroptosis [11]. This process contributes to a self-perpetuating cycle of inflammation, ferroptosis, and MMP activation within articular cartilage, ultimately exacerbating tissue destruction. Therefore, modulation of ferroptosis represents a promising therapeutic target for the prevention and treatment of KOA.

Traditional Chinese herbal medicine (CHM) formulations have historically played an essential therapeutic role in the management of KOA in China and have increasingly attracted global interest due to their demonstrated ability to reduce Visual Analog Scale, Western Ontario and McMaster Universities Osteoarthritis Index, and Lequesne index scores, while simultaneously improving Lysholm scores and overall treatment efficacy. These formulations have also been associated with favorable tolerability and a high safety profile [12–14]. 

Prior studies have reported that various plant-derived extracts and secondary metabolites exert beneficial effects in KOA by inhibiting the expression of inflammation- and matrix degradation-related factors, and by modulating multiple signaling pathways and cell death mechanisms [15]. CHM prescriptions, typically consisting of synergistic combinations of herbal ingredients enhance therapeutic efficacy and reduce adverse effects. However, the existing body of quantitative research on the clinical effectiveness of CHM in KOA remains limited, and the underlying molecular mechanisms have not been fully elucidated. Therefore, clarification of the mechanisms through which CHM exerts its therapeutic effects in KOA is of substantial scientific importance and is necessary to support its clinical application.

Bushen Chushi Formula (BSCSF) is a modified herbal prescription developed based on the classical Zuo Gui Pill from Jingyue Quanshu, formulated with the aim of tonifying the liver and kidney, eliminating dampness, and promoting the circulation of qi and blood. Clinical observations and prior investigations have indicated that BSCSF may alleviate joint pain, improve joint function in individuals with KOA, modulate inflammatory mediator levels, and inhibit degradation of the cartilage extracellular matrix [16, 17]. 

This study was designed to investigate the therapeutic effects of BSCSF in an animal model of KOA, focusing on dose-dependent outcomes related to cartilage preservation, enhancement of subchondral bone microarchitecture, and modulation of the ferroptosis-associated MAPK/SLC7A11/GPX4 signaling pathway. By integrating the theoretical principles of traditional Chinese medicine with contemporary molecular research methodologies, the study aims to provide scientific evidence supporting the evidence-based use of BSCSF in the treatment of KOA and other skeletal muscle diseases.

## Methods

### Animal grouping and medicine Preparation for Gavage

A total of 66 male Sprague-Dawley rats, aged 6–7 weeks with a mean body weight of 226.94 ± 7.90 g, were obtained from Beijing Charles River Laboratories (production license: SCXK (Beijing) 2021-0011). Animal housing and all experimental procedures were performed in accordance with the regulations established by the Experimental Animal Management Committee and were approved by the institutional ethics committee (ethical approval number: 2024B126), ensuring adherence to animal welfare standards.

The animals were randomly assigned into two initial groups: 13 rats were designated as the blank control group, and the remaining 53 rats underwent monosodium iodoacetate (MIA)-induced KOA modeling. Upon completion of the modeling process, 3 rats from the group that underwent modeling and 3 from the control group were randomly selected for preliminary evaluation to confirm the successful establishment of the KOA model. Following confirmation, the remaining 50 rats that underwent modeling were randomly divided into five groups (*n* = 10 per group): model group, diclofenac sodium enteric-coated tablets (DCF) group, and Bushen Chushi Formula high-dose (BSCSF-H), medium-dose (BSCSF-M), and low-dose (BSCSF-L) groups, and the control group contained the remaining 10 rats.

BSCSF was provided as a dry extract powder with a crude drug-to-extract ratio of 2.9:1 (w/w) by the Institute of Chinese Materia Medica, China Academy of Chinese Medical Sciences. Diclofenac Sodium Enteric-Coated Tablets, produced by Tianjin Shike Pharmaceutical Co., Ltd., contained 25 mg of diclofenac sodium per tablet (Approval Number: National Medical Product Approval No. H11021640).

### Animal model establishment

The KOA model was established through intra-articular injection of MIA. Anesthesia was induced via intramuscular injection of Zoletil 50^®^ (Virbac, 972LA) at a dosage of 0.12 mL/100 g body weight. The right knee joint area was shaved and sterilized three times using iodine solution. Intra-articular injection was carried out with a 1 mL syringe inserted through the lateral joint space of the patellar ligament while the knee was maintained in a flexed position.

Rats in the model, DCF, and BSCSF low-, medium-, and high-dose groups received a 50 µL intra-articular injection of sterile 0.9% normal saline containing 6 mg/kg MIA. Rats in the control group were administered 50 µL of sterile 0.9% sodium chloride solution. Following the injection, the knee joint was passively moved through multiple cycles of flexion and extension to promote even distribution of the injected solution.

Seven days after induction, three rats were randomly selected from both the model-induced group and the control group for evaluation using Mankin’s scoring system and micro-computed tomography (Micro-CT) analysis, in order to verify the successful establishment of the KOA model [18]. 

### Administration and tissue collection

Rats in the control and model groups were administered 0.9% normal saline by gavage at a dose of 2 mL/kg. For the BSCSF groups, the equivalent rat dosage for the BSCSF-M was calculated as 9.27 g/kg using the Meeh-Rubner formula. The BSCSF-H received 18.54 g/kg (2× the medium dose), and the BSCSF-L received 4.635 g/kg (0.5× the medium dose). Diclofenac sodium enteric-coated tablets were administered to rats at an equivalent dose of 13.5 mg/kg, dissolved in purified water and delivered by gavage at a volume of 10 mL/kg. All treatments were administered once daily for a duration of 4 weeks. (Table 1)

**Table 1:** Grouping and intervention measures

Group	Intervention Measures
Control	Normal saline gavage
Model	Normal saline gavage
BSCSF-L	BSCSF gavage at 4.64 g/kg (equivalent)
BSCSF-M	BSCSF gavage at 9.27 g/kg (equivalent)
BSCSF-H	BSCSF gavage at 18.54 g/kg (equivalent)
DCF	DCF gavage at 13.5 mg/kg (equivalent)

Tissue collection was carried out one day following the final intervention. Anesthesia was induced with pentobarbital. Blood was collected from the abdominal aorta and allowed to stand for 1 h prior to serum separation, after which the serum was stored for subsequent analysis. The right knee joint was harvested with approximately 2 cm of the diaphysis retained above and below the joint line. Following Micro-CT scanning, the femoral condyles were separated using bone rongeurs; the lateral condyle was fixed in 4% formaldehyde, while the medial condyle was preserved at − 80 °C for further examination.

### Assessment of general conditions in rats

Body weight measurements were obtained at baseline (initial assessment), following one week of adaptive feeding, one week after modeling induction, and at weekly intervals during the intervention period (weeks 1, 2, 3, and 4). Throughout the experimental period, general clinical conditions were continuously monitored, including the presence of piloerection, cyanosis, dyspnea, changes in spontaneous activity or gait, tremors, convulsions, abnormal bodily secretions, and excretory abnormalities.

Evaluation of the right knee joints was conducted using the Lequesne MG scoring system, which assesses four parameters: pain, gait impairment, range of motion, and joint swelling [19]. Scoring was independently performed by two blinded investigators, and the final score for each rat was calculated as the mean of the two assessments.

### Micro-CT assessment of subchondral bone in rat knee joints

Imaging was conducted using the PerkinElmer Quantum GX2 Micro-CT system under the following conditions: voltage set at 90 kV, a field of view of 36 mm, and scan mode configured for high-resolution three-dimensional reconstruction. Data analysis was carried out using Caliper Micro-CT Analysis Tools within the Analyze software suite. Prior to scanning, the imaging center was adjusted in preview mode.

Reconstruction of the acquired CT data was performed with a pixel size of 15 μm. Cross-sectional data of the femur were obtained by sectioning, with 101 slices captured along the z-axis. Bone microarchitecture analysis was applied, and corresponding bone parameters and map.obj data files were saved for subsequent analysis. All bone parameters were systematically organized and subjected to statistical evaluation.

The primary indices included in the analysis were bone volume fraction (BV/TV), bone surface-to-volume ratio (BS/BV), trabecular thickness (Tb.Th), trabecular number (Tb.N), trabecular separation (Tb.Sp), and connectivity density (Conn.Dn).

### Histopathological observation

Histopathological evaluation was conducted using hematoxylin and eosin (HE) staining, Safranin O-Fast Green staining, and the Mankin’s scoring system to assess structural and cellular changes, as well as cartilage degeneration. Tissue samples fixed in 4% formaldehyde were decalcified in 10% ethylenediaminetetraacetic acid buffer for a period of 4 weeks. Upon completion of decalcification, the tissues were dehydrated, embedded in paraffin, and sectioned into continuous 6 μm slices using a microtome.

Serial sections were stained using HE staining with modified Lillie-Mayer hematoxylin (Leagene, DH000) and eosin solution (Beijing Chemical Factory, 20150915), as well as Safranin O-Fast Green staining based on the improved cartilage staining kit protocol (Solarbio, G1371). These staining techniques were employed to observe the morphological structure of the cartilage tissue.

Evaluation of cartilage integrity was carried out using Mankin’s scoring system, based on Safranin O-Fast Green-stained sections. Parameters assessed included matrix staining intensity, chondrocyte density, cartilage matrix characteristics, and the continuity of the tidemark.

### Immunofluorescence staining

Antigen retrieval for cartilage tissue sections was conducted using citrate buffer solution (Proteintech, PR30001) via heat-induced epitope retrieval. Endogenous peroxidase activity was blocked by incubating the sections in 3% hydrogen peroxide solution, followed by phosphate-buffered saline (PBS) washes.

Detection of p38 MAPK expression was performed using the p38 MAPK Ready-To-Use IHC Kit (Proteintech, KHC0118). For finding other target proteins, the following primary antibodies were applied: SLC7A11 Polyclonal Antibody (Thermo Fisher Scientific, PA1-16893, 1:200), GPX4 Polyclonal Antibody (Proteintech, 30388-1-AP, 1:200), and MMP-13 Polyclonal Antibody (Proteintech, 18165-1-AP, 1:400). Sections were incubated at 37 °C for 60 min. After PBS washing, the sections were further incubated with a secondary antibody (ZSGB-BIO, PV-6001) at 37 °C for 30 min, followed by an additional PBS wash.

Diaminobenzidine (Solarbio, DA1010) was used as the chromogen and applied for 7 min. Sections were then rinsed with PBS. Nuclear counterstaining was carried out with hematoxylin, after which the sections were dehydrated, cleared, and mounted using neutral balsam. Positive immunostaining was identified as brown coloration under light microscopy.

Quantitative analysis of protein expression was conducted by measuring the mean optical density (MOD) values using Image-Pro Plus 6.0 software.

### Detection of serum levels of interleukin-1 beta (IL-1β), tumor necrosis factor-alpha (TNF-α), GSH, and cartilage Fe²⁺ content

Blood samples (5 mL) were collected from the abdominal aorta of the experimental rats. The samples were allowed to stand at room temperature for 1 h and subsequently centrifuged at 3000 rpm for 10 min to separate the serum. Serum concentrations of IL-1β and TNF-α were measured using enzyme-linked immunosorbent assay kits, in accordance with the manufacturers’ instructions. Following completion of the assay procedures, absorbance values were recorded at a wavelength of 450 nm using a microplate reader. The concentrations of IL-1β and TNF-α were calculated based on the generated standard curves to evaluate the inflammatory response.

In addition, serum levels of GSH were determined, and Fe²⁺ expression in cartilage tissue was quantified, following the protocols specified in the reduced GSH test kit (Solarbio, BC1175) and the tissue iron content assay kit (Solarbio, BC4355), respectively.

### Detection of p38MAPK, SLC7A11, GPX4, and MMP-13 mRNA expression in rat cartilage tissue using real-time fluorescent quantitative PCR (RT-qPCR)

Total RNA was extracted using the Trizol reagent method (Thermo Fisher Scientific, 15596026), and RNA concentrations were subsequently quantified. Reverse transcription was performed using the TransScript^®^ One-Step gDNA Removal and cDNA Synthesis SuperMix (TransGen Biotech, AE311) in a 20 µL reaction system within a PCR instrument. The reaction conditions included incubation at 42 °C for 15 min, followed by heating at 85 °C for 5 s. The synthesized complementary DNA (cDNA) was stored at 4 °C.

Quantitative real-time PCR (RT-qPCR) was carried out using a two-step protocol in a 20 µL reaction volume per well, consisting of 7.2 µL RNase-free water, 0.4 µL forward primer, 0.4 µL reverse primer, 10 µL 2× TranStart^®^ Top/Tip Green qPCR SuperMix, and 2 µL cDNA. Each sample was tested in duplicate. The amplification protocol included an initial denaturation at 94 °C for 30 s, followed by 45 cycles of denaturation at 94 °C for 5 s and annealing/extension at 60 °C for 30 s.

All primers were synthesized by Beijing Biomed Gene Technology Co., Ltd. (Supplementary Table 1). For relative quantification, GAPDH was used as the internal reference gene, and relative mRNA expression levels of the target genes were calculated using the 2^−△△Ct^ method. Statistical analysis was then conducted based on the calculated values.

### Statistical analysis

Statistical analyses were conducted using SPSS version 26.0. Graphs were generated using GraphPad Prism 8.4 (GraphPad, USA). Continuous variables were presented as mean ± standard deviation. One-way analysis of variance was applied for comparisons among multiple groups when the data met the assumptions of normality and homogeneity of variance. Post hoc pairwise comparisons were performed using Bonferroni correction methods. For data that did not follow a normal distribution, the Kruskal–Wallis H test was utilized. All statistical tests were two-sided, and a *p* < 0.05 was considered statistically significant.

## Results

### Body weight and Lequesne MG score of rats

**Fig. 1:**
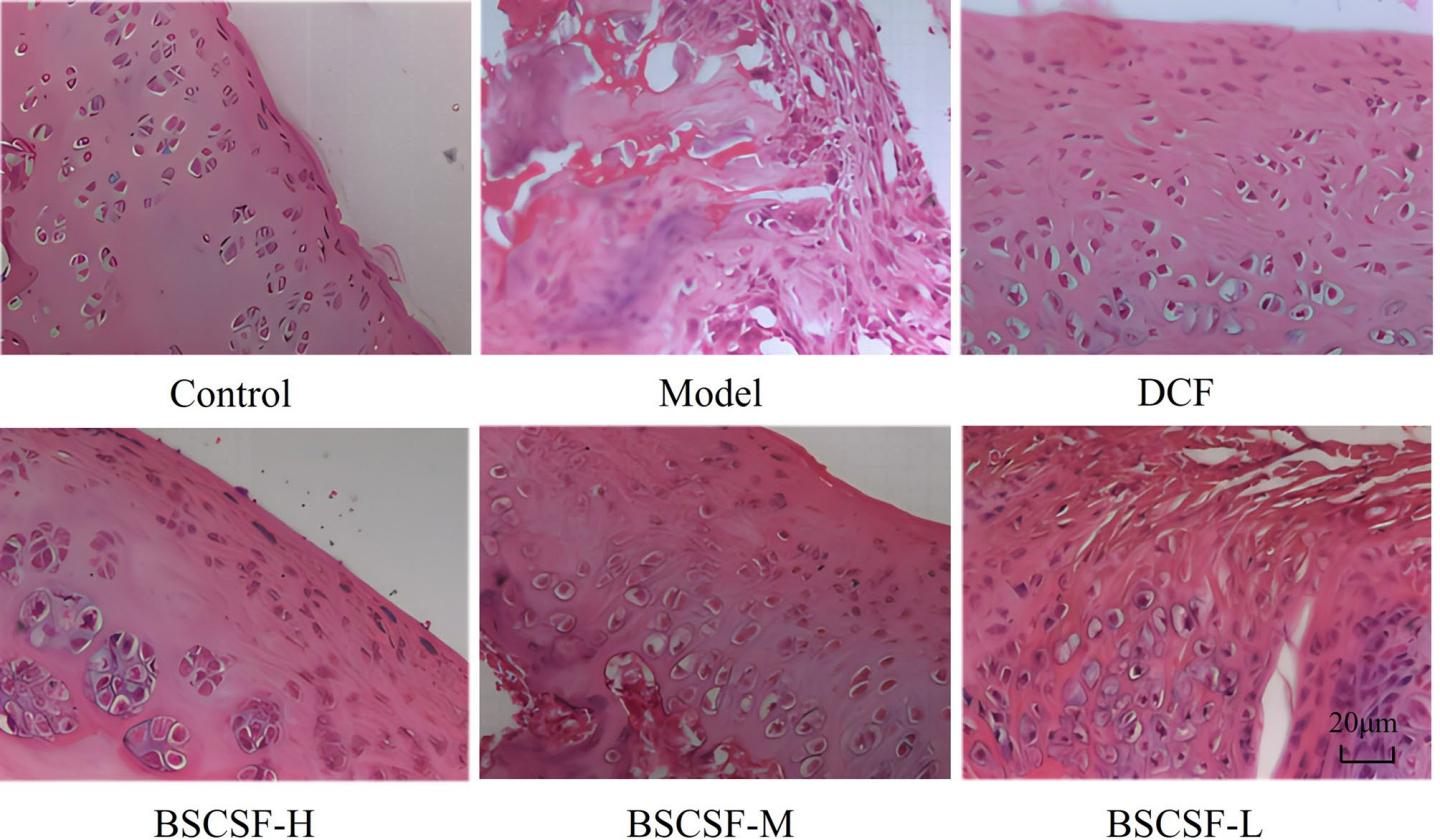
Effects of BSCSF on body weight and joint function in rats with knee osteoarthritis. (*n* = 10/group) (A) Changes in body weight across experimental groups over time (B) Comparison of Lequesne MG scores before and after intervention *Note*: Compared with the control group, #*p* < 0.05; pre-intervention vs. post-intervention within the same group, ^△^*p* < 0.05; DCF and BSCSF-H/M/L groups vs. model group, *p < 0.05; DCF and BSCSF-H/M groups vs. BSCSF-L group, ^a^*p* < 0.05

Body weight in all six groups indicated a gradual increase over time. No statistically significant differences in body weight were observed among the groups from the beginning of the experiment through the third week of drug intervention. However, by the fourth week, the body weight of the control group was higher than that of the other five groups (approximately 0.9-fold vs. control, *#p* < 0.05) (Table 2; Fig. 1A).

**Table 2:** Weight changes of rats in each group (g)

Group	Initial Body Weight	1-week after Adaptive Feeding	1-Week Post-Modeling	1-Week Intervention	2-Week Intervention	3-Week Intervention	4-Week Intervention
Control	229.85 ± 7.16	283.82± 8.36	328.29± 16.81	382.64± 19.23	417.17± 22.16	457.73± 26.71	486.25± 30.40
Model	226.71 ± 9.55	278.94± 14.64	327.43± 16.79	361.93± 25.22	380.35± 25.90	413.79± 30.38	434.40± 34.48^#^
DCF	224.99 ± 6.82	270.61± 14.43	317.25± 20.40	357.74± 17.72	382.00± 36.69	414.47± 50.86	431.11± 34.52^#^
BSCSF-H	224.88 ± 6.18	268.28± 10.52	318.69± 13.62	357.66± 27.75	393.51± 28.14	420.84± 30.49	436.09± 32.38^#^
BSCSF-M	224.53 ± 8.97	276.97± 15.43	326.55± 16.21	368.77± 23.18	385.61± 25.80	422.84± 29.01	440.20± 31.93^#^
BSCSF-L	231.19 ± 8.33	280.02± 14.85	339.62± 27.53	363.93± 20.29	392.79± 26.00	420.52± 31.57	437.35± 32.29^#^
F	1.286	1.97	1.77	1.743	2.36	2.317	4.290
*P*	0.284	0.098	0.135	0.141	0.052	0.056	0.002

Note: Other groups compared with control group ^#^*P* < 0.05

Throughout the observational period, rats in the control group exhibited a stable general condition, characterized by glossy fur, normal food intake, steady respiration, active spontaneous behavior, coordinated gait, and absence of visible knee joint swelling. In contrast, rats in the model group displayed signs of poor general condition, including slightly reduced food intake, rough and dull fur, accelerated breathing, decreased spontaneous activity, unsteady gait, and localized knee joint swelling. Following treatment, improvements in general condition and fur quality were observed in the BSCSF-L, BSCSF-M, BSCSF-H, and DCF groups, accompanied by reductions in knee joint swelling and normalization of gait and spontaneous activity.

At the end of the modeling phase, prior to intervention, Lequesne MG scores in the model, DCF, and BSCSF-H/M/L groups were significantly elevated compared to the control group (#*p* < 0.05). Following intervention, all four treatment groups presented significantly reduced Lequesne MG scores (^△^*p* < 0.05). Post-intervention scores in the DCF and BSCSF-H/M/L groups were significantly lower than those in the model group (*p < 0.05). Relative to the model group, Lequesne MG scores in the DCF, and BSCSF-H/M/L groups fell to 0.56-, 0.58-, 0.53-, and 0.73-fold, respectively. Additionally, the DCF and BSCSF-H/M groups demonstrated greater efficacy in score reduction compared to the BSCSF-L group (^a^*p* < 0.05) (Table 3; Fig. 1B).

**Table 3:** Comparison of Lequesne MG scores before and after intervention($\:\stackrel{-}{x\:}$± *s*, *n* = 10)

Group	Before intervention	After intervention
Control	0	0
Model	7.50 ± 1.08^#^	6.60 ± 1.65^#△^
DCF	7.20 ± 1.14^#^	3.70 ± 0.48^#△*a^
BSCSF-H	7.20 ± 1.87^#^	3.80 ± 0.79^#△*a^
BSCSF-M	7.30 ± 1.34^#^	3.50 ± 0.85^#△*a^
BSCSF-L	7.40 ± 0.70^#^	4.80 ± 0.79^#△*^

Other groups compared with control group ^#^*P* < 0.05

Comparison between pre-intervention and post-intervention within each group ^△^*P* < 0.05

DCM group and BSCSF-H/M/L groups compared with model group ^*^P < 0.05

DCM group and BSCSF-H/M groups compared with BSCSF-L group ^a^*P*<0.05

### Observation of subchondral bone in rat knee joint by micro-CT

**Fig. 2:**
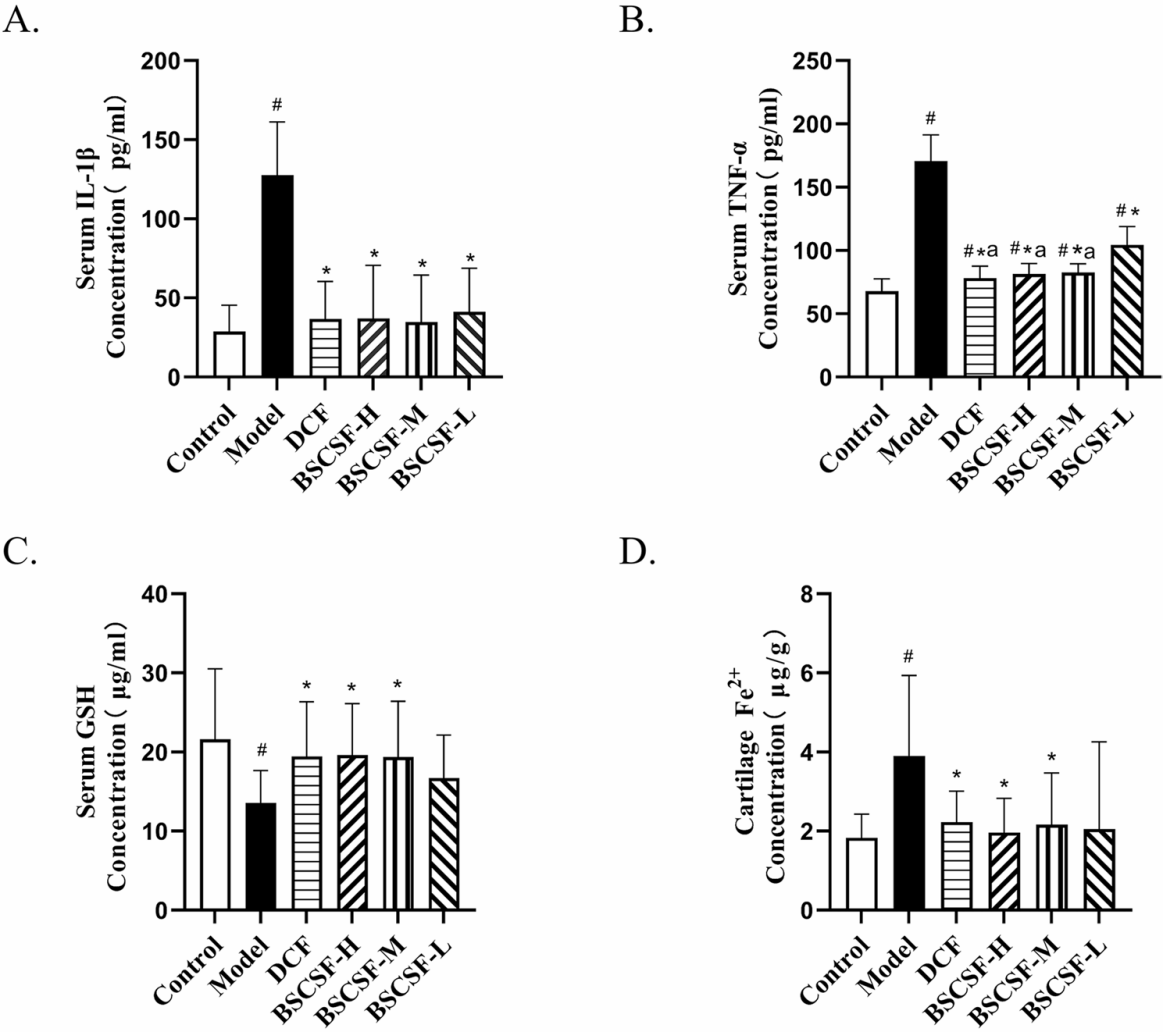
Representative micro-CT images of the rat knee joints in each group. (*n* = 8/group)

Micro-CT imaging of rat knee joints (Fig. 2) demonstrated that the control group exhibited well-preserved joint architecture, with regular morphology of the femoral condyles, tibial plateaus, and patellar articular surfaces. These surfaces were smooth, with no evidence of osteosclerosis or erosive lesions. The subchondral trabecular bone displayed clear structural organization and uniform bone mineral density distribution.

In contrast, the model group exhibited pronounced pathological changes, including marginal osteophyte formation, irregular articular surfaces of the femoral condyles and tibial plateaus, focal cartilage erosion with surface defects, osteogenic hyperplasia, cortical bone thinning, and rarefaction of the trabecular network. These changes were characterized by a reduction in trabecular number, discontinuity in trabecular architecture, and impaired structural alignment.

Following treatment, the DCF and BSCSF-H/M/L groups presented varying degrees of improvement in joint pathology compared to the model group. These improvements appeared dose-dependent and were marked by partial restoration of trabecular microarchitecture, as evidenced by increased trabecular thickness, enhanced trabecular connectivity, and improved structural organization.

**Fig. 3:**
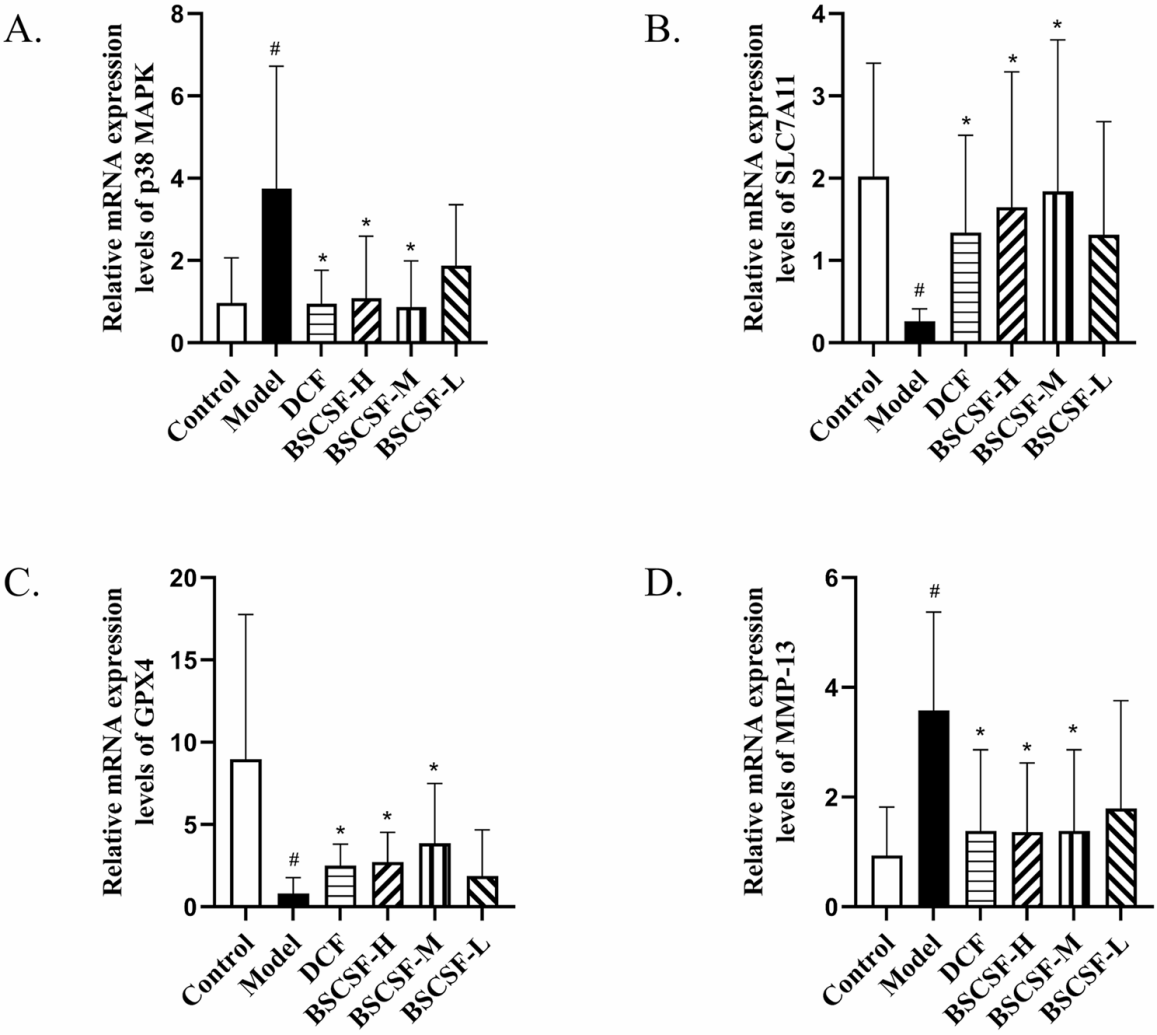
Quantitative analysis of subchondral bone microarchitecture in the knee joints. (*n* = 8/group) (**A**) Bone volume fraction (*BV*/*TV*), (**B**) Bone surface-to-volume ratio (*BS*/*BV*), (**C**) Trabecular separation (*Tb*.*Sp*), (**D**) Trabecular thickness (*Tb*.*Th*), (**E**) Trabecular number (*Tb*.*N*), (**F**) Connectivity density (*Conn*.*Dn*)

Quantitative analysis of bone parameters indicated that BV/TV and Tb.N in the model group decreased to 0.58-fold and 0.55-fold of the control group values (#*p* < 0.05), respectively, while both parameters were significantly increased in the BSCSF-M group (BV/TV: 0.98-fold vs. control, Tb.N: 1.01-fold vs. control) when compared to the model group (*p < 0.05). The BS/BV and Tb.Sp were elevated in the model group (approximately BS/BV: 1.17-fold; Tb.Sp: 1.35-fold) compared to the control group (#*p* < 0.05); both parameters were significantly reduced in the DCF (BS/BV: 1.04-fold vs. control; Tb.Sp: 1.12-fold vs. control) and BSCSF-M (BS/BV: 1.04-fold vs. control; Tb.Sp: 0.91-fold vs. control) groups compared to the model group (*p < 0.05). Tb.Th was significantly lower in the model group than in the control group, decreased to 0.75-fold (#*p* < 0.05), and significantly higher values were recorded in the DCF (1.00-fold vs. control), BSCSF-H (1.00-fold vs. control), and BSCSF-M (1.00-fold vs. control) groups compared to that in the model group (*p < 0.05). Analysis of Conn.Dn indicated a significant reduction in the model group when compared to the control group, decreased to 0.66-fold (#*p* < 0.05), while a significant increase was observed in the BSCSF-M group (1.03-fold vs. control) compared to the model group (*p < 0.05). Additionally, Conn.Dn was significantly lower in the DCF group (0.73-fold vs. control) compared to the BSCSF-M group (b*p* < 0.05) (Table 4; Fig. 3).

**Table 4:** Morphological indices of subchondral bone in the knee joints of rats($\:\stackrel{-}{x}$± s, *n* = 8)

Group	BV/TV(%)	BS/BV(mm^−1^)	Tb.Sp(mm)	Tb.Th(mm)	Tb.*N*(mm^−1^)	Conn.Dn(mm^−3^)
Control	23.69 ± 7.22	19.77± 1.88	0.89± 0.16	0.24± 0.06	0.65± 0.31	17.55± 1.41
Model	13.74 ± 6.83^#^	23.05± 1.86^#^	1.20± 0.17^#^	0.18± 0.03^#^	0.36± 0.21^#^	11.64± 6.91^#^
DCF	18.08 ± 11.47	20.53± 2.02^*^	1.00± 0.20^*^	0.24± 0.05^*^	0.42± 0.30	12.77± 7.46^b^
BSCSF-H	19.51 ± 11.78	21.65± 2.60	1.01± 0.32	0.24± 0.06^*^	0.46± 0.42	18.00± 12.42
BSCSF-M	23.31 ± 5.94^*^	20.54± 1.38^*^	0.81± 0.16^*^	0.24± 0.06^*^	0.66± 0.23^*^	20.94± 5.79^*^
BSCSF-L	18.54 ± 9.42	21.38± 1.61	1.03± 0.23	0.21± 0.07	0.42± 0.30	15.17± 8.75

Other groups compared with control group ^#^*P* < 0.05

DCM group and BSCSF-H/M/L groups compared with model group ^*^P < 0.05

DCM group and BSCSF-H/L groups compared with BSCSF-M group ^b^*P*<0.05

### Histopathological staining results

HE staining indicated that the articular cartilage in the control group exhibited a smooth surface with well-defined structural layers, normal chondrocyte morphology, and uniform cell distribution. In contrast, the model group demonstrated a rough cartilage surface with substantial structural destruction, increased fissures and fibrosis, reduced cartilage layer thickness, uneven staining, hypertrophic chondrocytes, apoptotic cells, and significant inflammatory cell infiltration in both cartilage and subchondral bone regions.

**Fig. 4:**
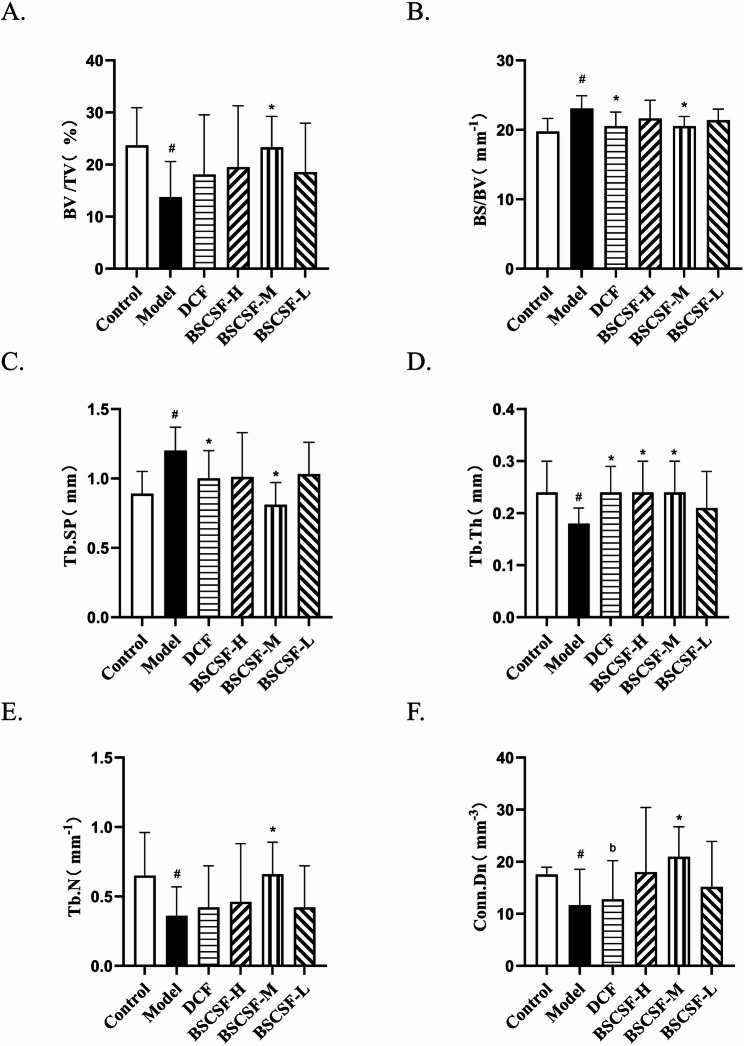
Comparison of hematoxylin and eosin (HE) stained sections of articular cartilage (×20 magnification). (*n* = 10/group)

In the DCF, BSCSF-H, and BSCSF-M groups, cartilage damage appeared milder compared to the model group. These groups presented relatively smoother cartilage surfaces, fewer fissures, improved fibrosis, better preservation of cartilage layer thickness, reduced chondrocyte disorganization and hypertrophy, and decreased inflammatory cell infiltration. In the BSCSF-L group, the cartilage surface was smoother than in the model group, although more pronounced fissures and fibrosis were observed when compared to the other treatment groups. This group also exhibited some hypertrophic chondrocytes and clustering, with better-preserved extracellular matrix compared to the model group, though inflammatory cell infiltration was still present and less favorably reduced than in the DCF and BSCSF-H/M groups (Fig. 4).

Safranin O–Fast Green staining results presented that in the control group, the cartilage surface remained intact and smooth, with uniform red staining of the matrix, blue-green staining of the subchondral bone, orderly chondrocyte arrangement, and no signs of fibrosis or hyperplasia. In the model group, the cartilage surface appeared rough and disrupted, with prominent fibrosis and fissures, a marked reduction in cartilage thickness, diminished red matrix staining, reduced chondrocyte number, disordered cellular arrangement, and evident inflammatory infiltration.

**Fig. 5:**
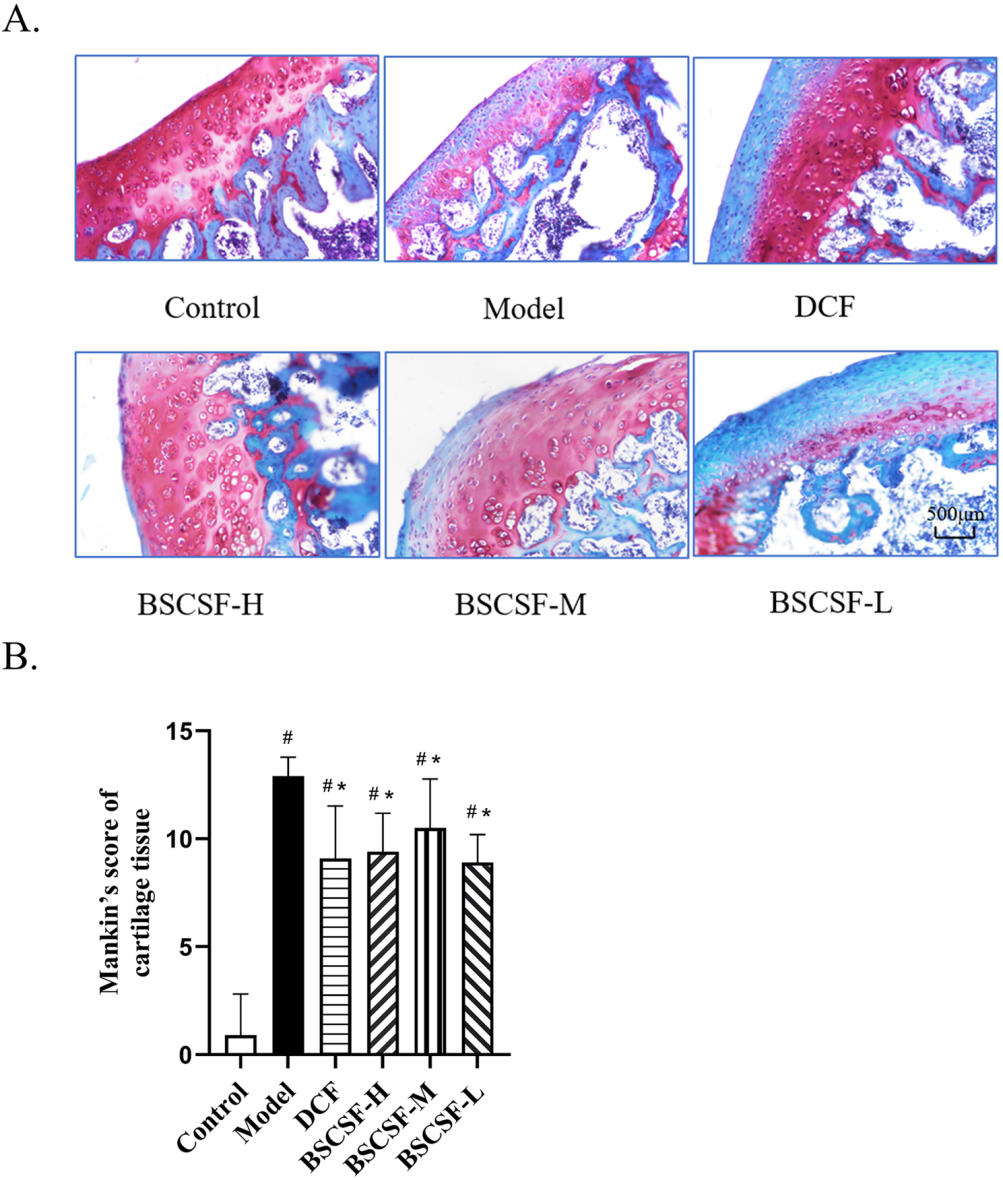
Effects of BSCSF on cartilage morphology and histopathological scores in rats with knee osteoarthritis. (*n* = 10/group) (**A**) Comparison of Safranin O–Fast Green staining of articular cartilage (×20 magnification). (**B**) Mankin’s scores of cartilage tissue *Note*: Compared with the control group, *#p* < 0.05; DCF and BSCSF-H/M/L groups vs. model group, *p < 0.05

Improved cartilage repair was observed in the DCF, BSCSF-H, and BSCSF-M groups compared to the model group. These improvements were reflected by increased retention of red matrix staining, greater cartilage thickness, reduced inflammatory infiltration, and enhanced chondrocyte number and alignment. The BSCSF-L group also exhibited an increase in cartilage thickness and improved chondrocyte number and organization when compared to the model group; however, the intensity of red matrix staining was lower than that observed in the other treatment groups (Fig. 5A).

Mankin’s scores were significantly increased in the model group (14.33-fold vs. control), DCF group (10.11-fold vs. control), and BSCSF-H (10.44-fold vs. control)/M (11.67-fold vs. control)/L (9.89-fold vs. control) groups compared to the control group (#*p* < 0.05). Following treatment, significantly lower scores were recorded in the DCF and BSCSF-H/M/L groups when compared to the model group (*p < 0.05) (Fig. 5B).

### Immunohistochemical results of p38 MAPK, SLC7A11, GPX4, and MMP-13 proteins in articular cartilage

The relative protein expression of p38 MAPK in articular cartilage, as indicated by MOD, was significantly elevated in the model group (1.91-fold) compared to the control group (#*p* < 0.05). In contrast, MOD values in the DCF (1.09-fold vs. control), BSCSF-H (1.09-fold vs. control), and BSCSF-M (1.09-fold vs. control) groups were significantly lower than those observed in the model group (*p < 0.05).

For SLC7A11, the MOD value in the model group showed a statistically significant decrease compared to the control group, to 0.59-fold of the control value (#*p* < 0.05). Following intervention, significantly higher MOD values were recorded in the DCF (0.89-fold vs. control), BSCSF-H (0.96-fold vs. control), and BSCSF-M (0.96-fold vs. control) groups when compared to the model group (*p < 0.05).

For GPX4, the MOD value was significantly reduced to 0.57-fold in the model group and to 0.65-fold in the BSCSF-L group relative to the control (#*p* < 0.05). However, the DCF (0.78-fold vs. control), BSCSF-H (0.86-fold vs. control), and BSCSF-M (0.97-fold vs. control) groups demonstrated significantly increasing MOD values when compared to the model group (*p < 0.05), while the BSCSF-M group indicating significantly higher expression than the BSCSF-L group (^a^*p*<0.05).

**Fig. 6:**
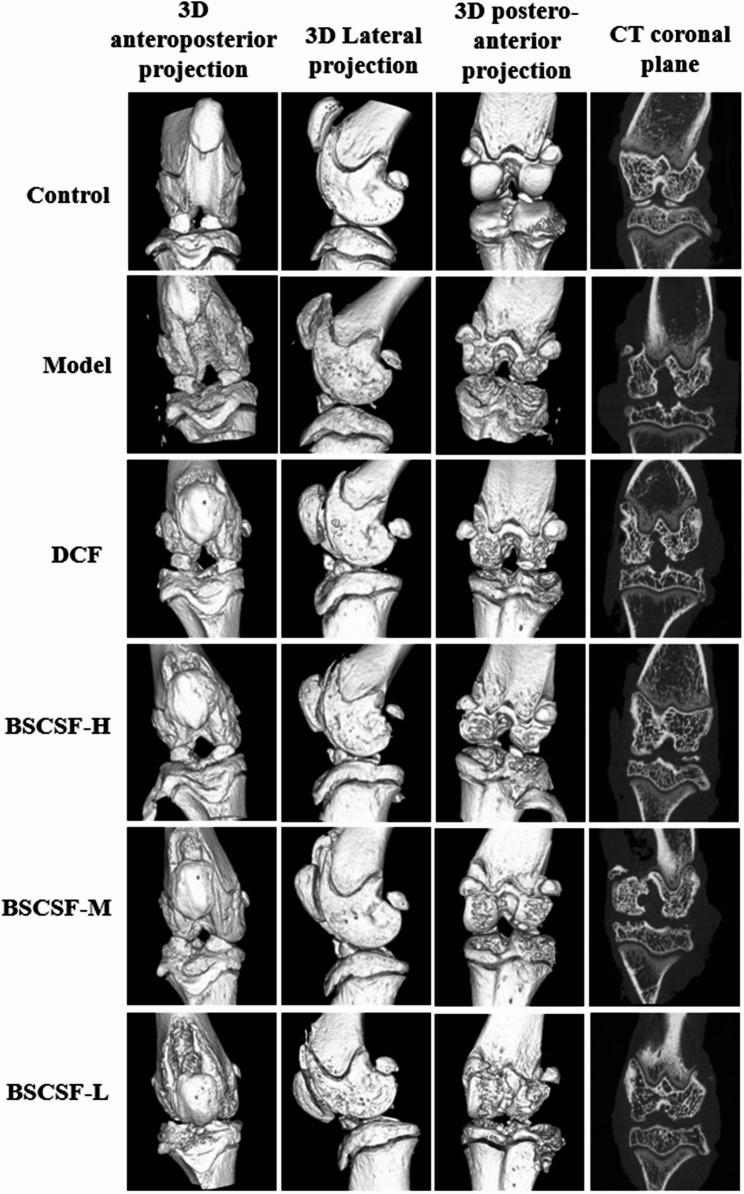
Immunohistochemical analysis of cartilage tissue. (*n* = 10/group) (**A**) Staining for p38 MAPK (×20), (**B**) MOD values of p38 MAPK, (**C**) Staining for SLC7A11 (×20), (**D**) Staining for GPX4 (×20), (**E**) MOD values of GPX4, (**F**) Staining for MMP-13 (×20), (**G**) Additional MMP-13 staining (×20), (**H**) MOD values of MMP-13 *Note*: Compared with the control group, *#p* < 0.05, *DCF *and BSCSF-H/M/L groups vs. model group, *p < 0.05, *DCF *and BSCSF-H/M groups vs. BSCSF-L group, ^a^*p* < 0.05

Analysis of MMP-13 expression indicated a significant increase in MOD in the model group (2-fold) and the BSCSF-H (1.55-fold)/M (1.45-fold)/L (1.73-fold) groups compared to the control group (#*p* < 0.05). Notably, the MOD values in the DCF (1.45-fold vs. control), BSCSF-H, and BSCSF-M groups were significantly lower than those in the model group (*p < 0.05) (Table 5; Fig. 6).

**Table 5:** The MOD of p38 MAPK, SLC7A11, GPX4, and MMP-13 ($\:\stackrel{-}{x}$± *s*, *n* = 10)

Group	p38 MAPK	SLC7A11	GPX4	MMP-13
Control	0.11 ± 0.04	0.27 ± 0.10	0.37 ± 0.17	0.11 ± 0.02
Model	0.21 ± 0.10^#^	0.16 ± 0.04^#^	0.21 ± 0.06^#^	0.22 ± 0.02^#^
DCF	0.12 ± 0.04^*^	0.24 ± 0.09^*^	0.29 ± 0.04^*^	0.16 ± 0.09
BSCSF-H	0.12 ± 0.04^*^	0.26 ± 0.13^*^	0.32 ± 0.10^*^	0.17 ± 0.05^#*^
BSCSF-M	0.12 ± 0.03^*^	0.26 ± 0.11^*^	0.36 ± 0.16^*a^	0.16 ± 0.07^#*^
BSCSF-L	0.14 ± 0.05	0.21 ± 0.07	0.24 ± 0.08^#^	0.19 ± 0.05^#^

Other groups compared with control group ^#^*P* < 0.05

DCM group and BSCSF-H/M/L groups compared with model group ^*^P < 0.05

DCM group and BSCSF-H/M groups compared with BSCSF-L group ^a^*P*<0.05

### Serum levels of IL-1β, TNF-α, and GSH and Fe²⁺ content in cartilage

Serum concentrations of IL-1β and TNF-α were significantly elevated in the model group (IL-1β: 4.43-fold; TNF-α: 2.51-fold) compared to the control group (#*p* < 0.05). Compared with the model group, the levels of IL-1β and TNF-α were significantly reduced in the DCF (IL-1β: 1.28-fold; TNF-α: 1.15-fold vs. control), BSCSF-H (IL-1β: 1.29-fold; TNF-α: 1.20-fold vs. control), BSCSF-M (IL-1β: 1.21-fold; TNF-α: 1.22-fold vs. control), and BSCSF-L (IL-1β:1.43-fold; TNF-α: 1.54-fold vs. control) groups (*p < 0.05). Additionally, TNF-α levels in the DCF, BSCSF-H, and BSCSF-M groups were significantly lower than those in the BSCSF-L group (^a^*p*<0.05).

A significant reduction was observed in the GSH content of the model group, to 0.63-fold of the control value (#*p* < 0.05). Significant increases in GSH levels were found in the DCF (0.90-fold vs. control), BSCSF-H (0.91-fold vs. control), and BSCSF-M (0.90-fold vs. control) groups when compared to the model group (*p < 0.05).

**Fig. 7:**
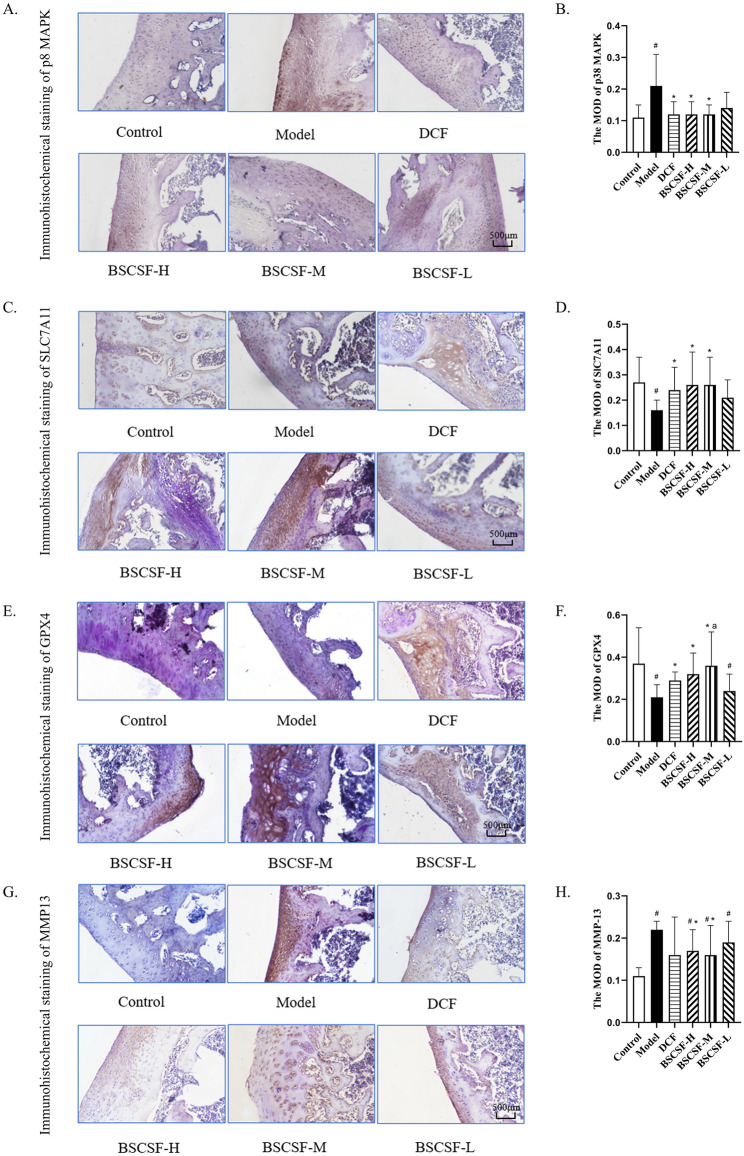
Effects of BSCSF on systemic inflammation, oxidative stress, and iron accumulation in rats with knee osteoarthritis. (*n* = 10/group, each sample measured in triplicate) (**A**) Serum IL-1β levels, (**B**) Serum TNF-α levels, (**C**) Serum glutathione (GSH) levels, (**D**) Cartilage Fe²⁺ content *Note*: Compared with the control group, *#p* < 0.05, DCF and BSCSF-H/M/L groups vs. model group, *p < 0.05; DCF and BSCSF-H/M groups vs. BSCSF-L group, ^a^*p* < 0.05

With respect to Fe²⁺ content in the cartilage of the knee joint, a significant elevation was observed in the model group (2.13-fold) compared to the control group (#*p* < 0.05). This increase was significantly attenuated in the DCF (1.22-fold vs. control), BSCSF-H (1.07-fold vs. control), and BSCSF-M (1.18-fold vs. control) groups when compared to the model group (*p < 0.05) (Table 6; Fig. 7).

**Table 6:** The contents of IL-1β, TNF-α, GSH in serum and Fe^2+^ in cartilage($\:\stackrel{-}{\text{x}}$± s, *n* = 10)

Group	IL-1β (pg/ml)	TNF-α (pg/ml)	GSH(µg/ml)	Fe^2+^(µg/g)
Control	28.80 ± 16.69	67.85 ± 9.73	21.60 ± 8.93	1.83 ± 0.60
Model	127.60 ± 33.61^#^	170.63 ± 20.72^#^	13.57 ± 4.09^#^	3.90 ± 2.04^#^
DCF	36.78 ± 23.60^*^	78.23 ± 9.27^#*a^	19.45 ± 6.90^*^	2.23 ± 0.78^*^
BSCSF-H	37.12 ± 33.52^*^	81.36 ± 8.30^#*a^	19.63 ± 6.49^*^	1.96 ± 0.87^*^
BSCSF-M	34.77 ± 29.59^*^	82.53 ± 6.91^#*a^	19.37 ± 7.06^*^	2.16 ± 1.31^*^
BSCSF-L	41.27 ± 27.47^*^	104.44 ± 14.34^#*^	16.71 ± 5.43	2.05 ± 2.21

Other groups compared with control group ^#^*P*< 0.05

DCM group and BSCSF-H/M/L groups compared with model group ^*^P< 0.05

DCM group and BSCSF-H/M groups compared with BSCSF-L group ^a^*P*<0.05

### Relative mRNA expression levels of p38 MAPK、SLC7A11、GPX4 and MMP-13 in cartilage

In cartilage tissue of the knee joint, the relative mRNA expression levels of p38 MAPK and MMP-13 were significantly elevated in the model group (p38 MAPK: 3.87-fold; MMP-13: 3.81-fold) compared to the control group (#*p* < 0.05). These expression levels were significantly reduced in the DCF (p38 MAPK: 0.98-fold; MMP-13: 1.47-fold vs. control), BSCSF-H (p38 MAPK: 1.11-fold; MMP-13: 1.45-fold vs. control), and BSCSF-M (p38 MAPK: 0.90-fold; MMP-13: 1.30-fold vs. control) groups when compared to the model group (*p < 0.05).

**Fig. 8:**
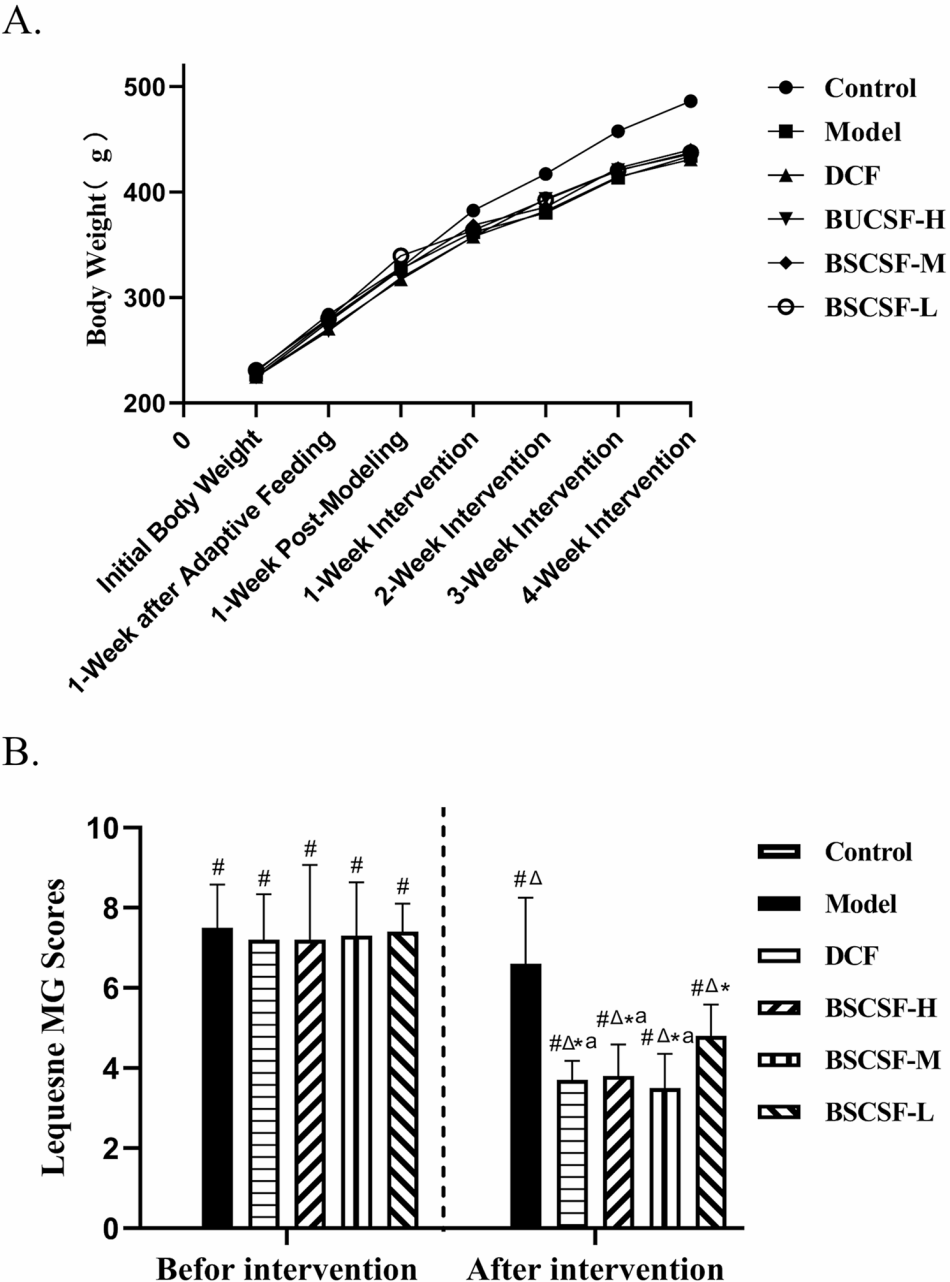
Effects of BSCSF on the expression of ferroptosis- and inflammation-related genes in cartilage tissue. (*n* = 10/group, each sample measured in triplicate) (**A**) Relative mRNA expression of p38 MAPK, (**B**) Relative mRNA expression of SLC7A11, (**C**) Relative mRNA expression of GPX4, (**D**) Relative mRNA expression of MMP-13 *Note*: Compared with the control group, *#p* < 0.05; DCF and BSCSF-H/M/L groups vs. model group, *p < 0.05

In contrast, the relative mRNA expression levels of SLC7A11 and GPX4 in the model group were significantly lower than those in the control group, reduced to 0.13-fold and 0.09-fold of the control levels, respectively (#*p* < 0.05), while significant increases in expression were observed in the DCF (SLC7A11: 0.66-fold; GPX4: 0.28-fold vs. control), BSCSF-H (SLC7A11: 0.82-fold; GPX4: 0.30-fold vs. control), and BSCSF-M (SLC7A11: 0.91-fold; GPX4: 0.43-fold vs. control) groups compared to the model group (*p < 0.05) (Table 7; Fig. 8).

**Table 7:** Relative mRNA expression levels of p38 MAPK、SLC7A11、GPX4、MMP-13 in cartilage($\:\stackrel{-}{x}$± *s*, *n* = 8)

Group	p38 MAPK	SLC7A11	GPX4	MMP-13
Control	0.97 ± 1.09	2.02 ± 1.38	8.98 ± 8.77	0.94 ± 0.88
Model	3.75 ± 2.97^#^	0.26 ± 0.15^#^	0.81 ± 0.97^#^	3.58 ± 1.79^#^
DCF	0.95 ± 0.81^*^	1.34 ± 1.18^*^	2.51 ± 1.29^*^	1.38 ± 1.48^*^
BSCSF-H	1.08 ± 1.51^*^	1.65 ± 1.64^*^	2.73 ± 1.79^*^	1.36 ± 1.26^*^
BSCSF-M	0.87 ± 1.12^*^	1.84 ± 1.84^*^	3.85 ± 3.64^*^	1.22 ± 1.11^*^
BSCSF-L	1.88 ± 1.48	1.31 ± 1.38	1.88 ± 2.80	1.79 ± 1.97

Other groups compared with control group ^#^*P* < 0.05

DCM group and BSCSF-H/M/L groups compared with model group ^*^P < 0.05

## Discussion

The BSCSF has been applied in the clinical management of KOA for several decades, with substantial clinical experience accumulated over time. Clinical practice has demonstrated that BSCSF possesses notable prophylactic and therapeutic efficacy in patients with KOA. This study further examined the potential mechanisms underlying the therapeutic effects of BSCSF, focusing on its modulation of ferroptosis-related pathways. Particular attention was given to its regulatory influence on inflammatory responses, cartilage matrix degradation, and pathological alterations in subchondral bone. Additionally, the therapeutic outcomes associated with different dosages were evaluated to comprehensively assess the overall efficacy of the formulation.

BSCSF reduces inflammatory responses and cartilage matrix damage in KOA. Knee osteoarthritis is characterized as an inflammatory osteoarticular condition, with interleukin-1β (IL-1β) and TNF-α recognized as key inflammatory mediators in its pathogenesis [20]. IL-1β promotes chondrocyte apoptosis and inhibits the synthesis of cartilage matrix components, whereas TNF-α exacerbates joint inflammation and cartilage damage by stimulating the production of inflammatory mediators like nitric oxide and prostaglandin E2 [21, 22]. In this study, serum levels of IL-1β and TNF-α were significantly elevated in the model group compared to the control group, reflecting the inflammatory cytokine response associated with KOA progression and confirming the successful establishment of the disease model.

Previous studies have reported that 5-hydroxymethylfurfural, a constituent of *Rehmannia glutinosa*, exerts anti-inflammatory and macrophage-protective effects by significantly downregulating both mRNA and protein levels of IL-1β and TNF-α in macrophage-like RAW 264.7 cells following pretreatment [23, 24]. Similarly, stigmasterol, a phytosterol component derived from *Achyranthes bidentata*, inhibits the release of pro-inflammatory cytokines, reduces cartilage matrix degradation, and suppresses IL-1β-induced activation of the NF-κB signaling pathway. It may also enhance the protective effects of ferroptosis inhibitors against IL-1β-mediated chondrocyte injury [25, 26]. 

In this study, significant reductions in IL-1β and TNF-α levels were observed in the BSCSF-H, BSCSF-M, BSCSF-L, and DCF groups compared to the model group, indicating that BSCSF exerts anti-inflammatory effects in the context of KOA. Notably, the BSCSF-H and BSCSF-M groups demonstrated anti-inflammatory efficacy comparable to that of the DCF group, both of which were more effective than the BSCSF-L group. These findings suggest that the anti-inflammatory activity of BSCSF may be dose-dependent, influenced by the concentration and combination of its herbal constituents.

Matrix metalloproteinase-13 (MMP-13) is a key enzyme involved in the degradation of type II collagen and plays a key role in the pathogenesis of cartilage degenerative diseases [27]. Overexpression of MMP-13 leads to the degradation of type II collagen and proteoglycans, thereby accelerating cartilage damage [28]. In this study, the BSCSF-H and BSCSF-M groups significantly downregulated both protein and mRNA expression levels of MMP-13, contributing to the preservation of cartilage structure by reducing matrix degradation in KOA.

Recent studies have confirmed the involvement of MAPKs and their receptor tyrosine kinases in the pathogenesis of osteoarthritis [29]. As a key component of the MAPK signaling pathway, p38 MAPK regulates cellular stress responses, inflammatory processes, and apoptosis, and plays a pivotal role in modulating inflammation and ferroptosis in KOA [30]. Ferroptosis-induced ROS can further activate the p38 MAPK pathway, promoting the overexpression of MMP-13.

In this study, the BSCSF-H, BSCSF-M, and DCF groups significantly downregulated p38 MAPK expression, accompanied by reduced expression levels of IL-1β, TNF-α, and MMP-13. These findings indicate that BSCSF may mitigate the stress response and pro-inflammatory gene expression in chondrocytes by inhibiting MAPK pathway activity. Through multi-pathway regulation of chondrocyte ferroptosis, BSCSF may contribute to the attenuation of chondrocyte damage and degeneration.

Furthermore, Safranin O–Fast Green staining demonstrated greater retention of red-stained cartilage matrix in the BSCSF-H and BSCSF-M groups, indicating reduced cartilage matrix degradation. Collectively, these findings propose that BSCSF may reduce serum IL-1β and TNF-α levels, suppress the inflammatory response within cartilage tissue, downregulate MMP-13 expression, improve the joint microenvironment, and attenuate cartilage damage.

### Potential protective effects of BSCSF on chondrocytes through modulation of ferroptosis

Ferroptosis in KOA is characterized by multidimensional pathological activation, including: (1) disruption of iron homeostasis leading to Fe²⁺ overload, which catalyzes the Fenton reaction and results in the generation of hydroxyl radicals; (2) peroxidation of polyunsaturated fatty acid-containing phospholipids, accompanied by ultrastructural damage and failure of PLOOH clearance due to impairment of the GPX4 antioxidant defense system; and (3) abnormal intracellular lipid accumulation [31, 32]. Ferroptosis not only contributes to chondrocyte injury but also accelerates the pathological progression of KOA by inducing inflammatory responses and promoting cartilage matrix degradation [33]. 

Upregulation of SLC7A11 enhances GSH synthesis, and concurrent upregulation of GPX4 increases the capacity of chondrocytes to counteract iron-dependent lipid peroxidation. This dual mechanism contributes to the inhibition of ferroptosis and the preservation of cell viability [34]. Rehmannioside A, an active compound derived from *Rehmannia glutinosa* Libosch, and a key pharmacologically active constituent in several traditional Chinese medicine (TCM) formulations used for conditions associated with “kidney deficiency,” has been reported to exert regulatory effects by significantly upregulating SLC7A11 expression and activating the SLC7A11/GPX4 signaling axis [35]. 

In this study, significant upregulation of SLC7A11 and GPX4 protein and mRNA expression levels, along with increased serum GSH concentrations, was observed in the BSCSF-H, BSCSF-M, and DCF groups compared to the model group. These changes were accompanied by a marked reduction in Fe²⁺ accumulation within cartilage tissue. Collectively, these findings indicate that BSCSF exerts protective effects against chondrocyte ferroptosis and contributes to the preservation of cartilage structural integrity and function through the upregulation of SLC7A11, GSH, and GPX4, as well as the reduction of intra-cartilaginous Fe²⁺ content.

### Improvement of cartilage and subchondral bone by BSCSF

A comprehensive histological evaluation of rat articular cartilage was conducted in this study. The model group exhibited characteristic pathological features of KOA, including an uneven cartilage surface, disorganized chondrocyte distribution, exposed subchondral bone, and diminished matrix staining. These alterations were reflected by a significantly elevated Mankin’s score, indicating pronounced structural joint damage. In contrast, the BSCSF-H, BSCSF-M, BSCSF-L, and DCF groups demonstrated notable therapeutic effects, as evidenced by improved cartilage architecture, smoother articular surfaces, reduced subchondral bone exposure, and more uniform matrix staining. The corresponding reductions in Mankin’s scores further confirmed that BSCSF effectively mitigated cartilage degeneration and contributed to the delay in KOA progression.

In recent years, Micro-CT has been recognized as the gold standard for assessing bone microarchitecture in small animal models [36]. When combined with specialized image analysis software, this technique allows for detailed morphological assessment of both cortical and trabecular bone structures [37]. In this study, the model group indicated significantly reduced values for BV/TV, Tb.Th, Tb.N, and Conn.Dn, along with increased BS/BV and Tb.Sp, when compared to the control group. These findings reflect deterioration in bone quality and disruption of the trabecular network, consistent with pathological changes observed in KOA. This degeneration is likely associated with localized inflammation, altered joint biomechanics, and dysregulated bone metabolism.

When compared to the model group, the DCF group demonstrated significant increases in Tb.Th and reductions in BS/BV and Tb.Sp, indicating improved bone matrix metabolism and enhanced trabecular mechanical properties, potentially through promotion of osteogenesis and inhibition of osteoclastic activity. The BSCSF-H group also presented significant increase in Tb.Th, indicating a beneficial effect at high dosage on trabecular structure and osteoclast regulation. Notably, the BSCSF-M group exhibited significant improvements across multiple parameters, including BV/TV, Tb.Th, Tb.N, and Conn.Dn, as well as decreases in BS/BV and Tb.Sp. These findings indicate that medium-dose BSCSF intervention exerted a comprehensive restorative effect on subchondral bone microarchitecture, particularly in enhancing trabecular connectivity density, thereby supporting the integrity of the bone network. These results underscore the importance of addressing both cartilage and subchondral bone in the treatment of KOA.

With regard to symptomatic improvement, Lequesne MG scores were significantly lower in all treatment groups compared to the model group. Moreover, scores in the DCF, BSCSF-H, and BSCSF-M groups were significantly lower than those in the BSCSF-L group, indicating more pronounced symptom relief and therapeutic efficacy. These outcomes are consistent with the histopathological and Micro-CT findings, indicating that the preservation of cartilage and subchondral bone structural integrity is closely associated with symptomatic improvement in KOA.

In conclusion, the results support the dual therapeutic role of BSCSF in maintaining cartilage structure and optimizing subchondral bone microarchitecture, thereby contributing to the attenuation of KOA-related symptoms.

### Optimal dosage and therapeutic advantages of BSCSF

Experimental data from this study indicated that the BSCSF-L group exhibited significant improvements over the model group only in Lequesne MG scores, Mankin’s scores, and protein expression levels of IL-1β and TNF-α. These findings suggest that BSCSF-L effectively alleviated inflammatory responses, reduced joint cartilage damage to a limited extent, and improved KOA symptoms. However, no significant regulatory effects on ferroptosis-related pathway molecules or subchondral bone structure were observed at this dosage.

In contrast, the DCF group and the BSCSF-H and BSCSF-M groups demonstrated favorable regulatory effects on inflammatory markers as well as the MAPK/SLC7A11/GPX4 signaling axis. Notably, the BSCSF-M group exhibited a trend toward greater upregulation of SLC7A11 and GPX4 at both the protein and mRNA levels, although these differences did not reach statistical significance.

Micro-CT analysis indicated that the BSCSF-M group indicated statistically significant improvements in multiple bone microstructural parameters compared to the model group. Specifically, Conn.Dn was significantly higher in the BSCSF-M group than in the DCF group. Additionally, BV/TV and Tb.N demonstrated an increasing trend when compared to other intervention groups, though these differences were not statistically significant. The BSCSF-H group indicated a significant increase only in Tb.Th.

Prior studies indicated that ferroptosis influences the function of bone marrow-derived mesenchymal stem cells, thereby affecting osteoblast and osteoclast activity, and has been identified as a novel therapeutic target for osteoporosis and osteoarthritis [38, 39]. The dose-dependent differences observed in the current study may be attributed to varying concentrations of TCM components influencing ferroptosis, osteogenesis, and osteoclastogenesis. The BSCSF-M group, in particular, demonstrated potential advantages in regulating subchondral bone metabolism, promoting osteogenesis, and improving bone microarchitecture in KOA.

These findings propose that medium-dose BSCSF is sufficient to exert significant therapeutic efficacy, as evidenced by the activation of anti-ferroptosis pathways and anti-inflammatory responses, ultimately contributing to cartilage and subchondral bone protection. Although high-dose BSCSF did not present a statistically significant enhancement in therapeutic outcomes, further investigation is warranted to assess its stability and consistency in maintaining efficacy, which may be important for dosage optimization in the treatment of cartilage degenerative diseases.

The multi-targeted effects of BSCSF may be attributable to the multi-pathway pharmacological actions of its compound TCM constituents, including anti-inflammatory, antioxidant, and bone remodeling regulatory mechanisms. Unlike modern pharmacological agents that typically act via single-target pathways, BSCSF may exert synergistic multi-target effects that rapidly inhibit ferroptosis and cartilage degeneration, thereby potentially delaying KOA progression through sustained improvements in the cartilage and subchondral bone microenvironment [40]. 

## Conclusion

BSCSF upregulates the expression of ferroptosis inhibitors SLC7A11 and GPX4 and increases GSH levels, thereby mitigating ferroptosis-induced damage to chondrocytes. The expression levels of IL-1β, TNF-α, MMP-13, and p38 MAPK were downregulated, resulting in attenuation of inflammatory responses and a reduction in extracellular matrix degradation in chondrocytes. Regulation of the ferroptosis process in chondrocytes was mediated through the MAPK/SLC7A11/GPX4 signaling axis, contributing to chondrocyte protection and delayed cartilage degeneration. Additionally, BSCSF demonstrated a capacity to modulate the subchondral bone microenvironment. Collectively, these effects contributed to the inhibition of KOA progression and alleviation of KOA-associated symptoms.

## Supplementary Information

Supplementary Material 1.

## Data Availability

All data generated or analysed during this study are included in this article. Further enquiries can be directed to the corresponding author.

## Abbreviations

BSCSF: Bushen Chushi Formula
DCF: diclofenac sodium
KOA: Knee osteoarthritis
PLOOH: Phospholipid hydroperoxides
GPX4: Glutathione peroxidase 4
SLC7A11: Solute carrier family 7 member 11
MMPs: Matrix metalloproteinases
MAPK: Mitogen-activated protein kinase
CHM: Chinese Herbal Medicine
VAS: Visual Analog Scale
WOMAC: Western Ontario and McMaster Universities Osteoarthritis Index
BV/TV: Bone volume fraction
BS/BV: Bone surface-to-volume ratio
Tb.Th: Trabecular thickness
Tb.N: Trabecular number
Tb.Sp: Trabecular separation
Conn.Dn: Connectivity density
HE: Hematoxylin and eosin
PBS: Phosphate-buffered saline
MOD: Mean optical density
IL-1β: Interleukin-1 beta
TNF-α: Tumor necrosis factor-alpha
RT-qPCR: Real-time fluorescent quantitative PCR
NO: Nitric oxide
PGE2: Prostaglandin E2
5-HMF: 5-Hydroxymethylfurfural
ROS: Reactive oxygen species
TCM: Traditional Chinese medicine
