# Supplementary material for: Therapeutic effects of Bushen Chushi formula on knee osteoarthritis via modulation of MAPK/SLC7A11/GPX4 signaling in rats
Source: Hereditas. 2025 Oct 29;162:220. doi: 10.1186/s41065-025-00587-1 (PMC12573852; doi:10.1186/s41065-025-00587-1)
Supplement: Supplementary file 1 — Supplementary Material 1. [file 41065_2025_587_MOESM1_ESM.doc]

Supplementary Table 1 Primer sequences for RT-qPCR

| Gene | F/R | Primer sequence (5' to 3') |
| --- | --- | --- |
| p38MAPK | Forward | TCAGGCTCTTCCATTCGTCT |
|  | Reverse | AGGAGAGGCCCACGTTCTAC |
| SLC7A11 | Forward | GACTGCCTTGACTTCCGTGA |
|  | Reverse | CAACGCTGTCTCTCACTGGT |
| GPX4 | Forward | GGTTACTGGGACCTAGGGGA |
|  | Reverse | GGGGACAAAGAGCCGGTAG |
| MMP-13 | Forward | CTCTGGTGTTTTGGGGTGCT |
|  | Reverse | CCCTGGAGCCCTGATGTTT |
| GAPDH | Forward | GCACCGTCAAGGCTGAGA |
|  | Reverse | AGCATCGCCCCACTTGATT |
